# 6-(4-Methyl­phen­yl)-3-(3,4,5-trimethoxy­phen­yl)-1,2,4-triazolo[3,4-*b*][1,3,4]thiadiazole

**DOI:** 10.1107/S1600536808030936

**Published:** 2008-09-30

**Authors:** Hai-Tang Du, Hai-Jun Du, Weiyi Zhou

**Affiliations:** a Institute of Natural Products, Research Center for Eco-Environmental Sciences, Guiyang College, Guiyang 550005, People’s Republic of China; b School of Chemistry and Environment Science, Guizhou University for Nationalities, Guiyang 550025, People’s Republic of China; c Analytical Center, Tianjin University, Tianjin 300072, People’s Republic of China

## Abstract

In the mol­ecule of the title compound, C_19_H_18_N_4_O_3_S, the central heterocylic ring system is oriented with respect to the trimethoxy­phenyl and 4-methyl­phenyl rings at dihedral angles of 1.1 (5) and 15.1 (5)°, respectively. The dihedral angle between the two benzene rings is 16.1 (4)°. In the crystal structure, mol­ecules are linked by inter­molecular C—H⋯O hydrogen bonds, and an intra­molecular C—H⋯N inter­action also occurs.

## Related literature

For general background, see: Karabasanagouda *et al.* (2007 ▶); Mathew *et al.* (2007 ▶).
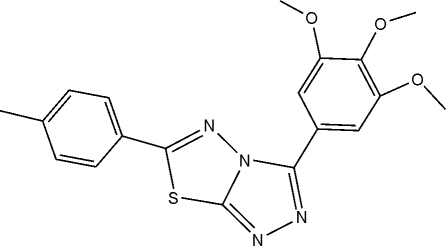


## Experimental

### 

#### Crystal data

C_19_H_18_N_4_O_3_S
*M* *_r_* = 382.43
Monoclinic, 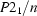

*a* = 7.2613 (15) Å
*b* = 18.519 (4) Å
*c* = 13.314 (3) Å
β = 100.52 (3)°
*V* = 1760.2 (6) Å^3^
*Z* = 4
Mo *K*α radiation
μ = 0.21 mm^−1^
*T* = 113 (2) K
0.20 × 0.18 × 0.12 mm

#### Data collection

Rigaku Saturn diffractometer
Absorption correction: multi-scan (*CrystalClear*; Rigaku/MSC, 2005 ▶) *T*_min_ = 0.959, *T*_max_ = 0.975
12668 measured reflections
4184 independent reflections
3593 reflections with *I* > 2σ(*I*)
*R*_int_ = 0.030

#### Refinement

*R*[*F* ^2^ > 2σ(*F* ^2^)] = 0.034
*wR*(*F* ^2^) = 0.094
*S* = 1.06
4184 reflections
248 parameters
H-atom parameters constrained
Δρ_max_ = 0.34 e Å^−3^
Δρ_min_ = −0.26 e Å^−3^

### 

Data collection: *CrystalClear* (Rigaku/MSC, 2005 ▶); cell refinement: *CrystalClear*; data reduction: *CrystalClear*; program(s) used to solve structure: *SHELXS97* (Sheldrick, 2008 ▶); program(s) used to refine structure: *SHELXL97* (Sheldrick, 2008 ▶); molecular graphics: *SHELXTL* (Sheldrick, 2008 ▶); software used to prepare material for publication: *SHELXTL*.

## Supplementary Material

Crystal structure: contains datablocks I, global. DOI: 10.1107/S1600536808030936/bx2183sup1.cif

Structure factors: contains datablocks I. DOI: 10.1107/S1600536808030936/bx2183Isup2.hkl

Additional supplementary materials:  crystallographic information; 3D view; checkCIF report

## Figures and Tables

**Table 1:** Hydrogen-bond geometry (Å, °)

*D*—H⋯*A*	*D*—H	H⋯*A*	*D*⋯*A*	*D*—H⋯*A*
C2—H2⋯N4	0.93	2.37	3.059 (2)	131
C7—H7*B*⋯O3^i^	0.96	2.45	3.202 (2)	135

Symmetry code: (i) 
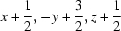
.

**Fig. 1.**
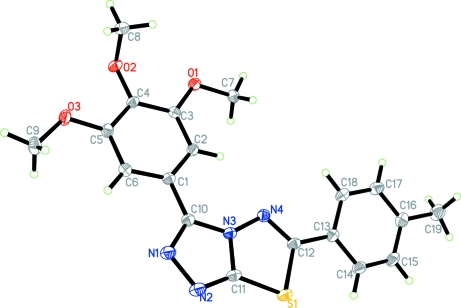
The molecular structure of the title molecule, with the atom-numbering scheme. Displacement ellipsoids are drawn at the 50% probability level.

**Fig. 2.**
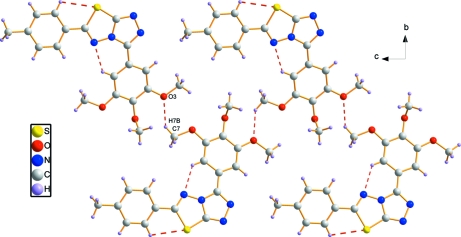
A partial packing diagram of the title compound. Hydrogen bonds are shown as dashed lines. [Symmetry code: O3 (x+1/2, -y+3/2, z+1/2)].

## supplementary crystallographic information

### Comment

1,2,4-Triazole and 1,3,4-thiadiazole represent one of the most biologically active classes of compounds, possessing a wide spectrum of activities. Various substituted 1,2,4-triazolo[3,4-*b*]-1,3,4-thiadiazoles are associated with diverse pharmacological activities such as antimicrobial (Karabasanagouda *et al.*, 2007) and anti-inflammatory activity (Mathew *et al.*, 2007). We report herein the crystal structure of the title compound.

In the molecule of the title compound (Fig. 1) the bond lengths and angles are within normal ranges. Rings A (C1—C6), B (N1—N3/C10/C11), C (S1/N3/N4/C11/C12) and D (C13—C18) are, of course, planar, and the dihedral angles between them are A/B = 0.61 (3)°, A/C = 1.57 (3)°, A/D = 16.09 (4)°, B/C = 0.96 (4)°, B/D = 15.53 (4)° and C/D = 14.60 (4)°. So, rings B and C are nearly coplanar. The coplanar ring system is oriented with respect to rings A and D at dihedral angles of 1.36 (3)° and 14.77 (4)°.

In the crystal structure, intramolecular C—H···N hydrogen bond results in the formation of a six-membered ring, intermolecular C—H···O hydrogen bonds link the molecules (Fig. 2), in which they may be effective in the stabilization of the structure.

### Experimental

For the preparation of the title compound, 4-amino-5-(3,4,5-trimethoxyphenyl) -4*H*-1,2,4-triazole-3-thiol (0.01 *M*) and 4-methylbenzoic acid (0.01 *M*) were dissolved in dry phosphorous oxychloride (10 ml). The resulted solution was further heated under reflux for 7 h. The reaction mixture was cooled to room temperature and the mixture was gradually poured onto crushed ice with stirring. Finally, powdered potassium carbonate and the required amount of solid potassium hydroxide were added until the pH of the mixture was raised to 8, to remove the excess of phosphorous oxychloride. The mixture was allowed to stand overnight and the solid was separated. It was filtered, washed with cold water, and then dried. Crystals suitable for X-ray analysis were obtained by the recrystallization of the solid residue from a mixture of *N*,*N*-dimethylformamide/ ethanol (1:1) by slow evaporation at room temperature.

### Refinement

H atoms were positioned geometrically, with C—H = 0.93 and 0.96 Å for aromatic and methyl H, respectively, and constrained to ride on their parent atoms with *U*_iso_(H) = *xU*_eq_(C), where *x* = 1.5 for methyl H and *x* = 1.2 for aromatic H atoms.

### Crystal data

**Table:**

C_19_H_18_N_4_O_3_S	*F*(000) = 800
*M**_r_* = 382.43	*D*_x_ = 1.443 Mg m^−^^3^
Monoclinic, *P*2_1_/*n*	Melting point: 461 K
Hall symbol: -P 2yn	Mo *K*α radiation, λ = 0.71073 Å
*a* = 7.2613 (15) Å	Cell parameters from 4938 reflections
*b* = 18.519 (4) Å	θ = 2.2–27.9°
*c* = 13.314 (3) Å	µ = 0.21 mm^−^^1^
β = 100.52 (3)°	*T* = 113 K
*V* = 1760.2 (6) Å^3^	Prism, colourless
*Z* = 4	0.20 × 0.18 × 0.12 mm

### Data collection

**Table:**

Rigaku Saturn diffractometer	4184 independent reflections
Radiation source: rotating anode	3593 reflections with *I* > 2σ(*I*)
confocal	*R*_int_ = 0.030
ω scans	θ_max_ = 27.9°, θ_min_ = 2.2°
Absorption correction: multi-scan (CrystalClear; Rigaku/MSC, 2005)	*h* = −9→9
*T*_min_ = 0.959, *T*_max_ = 0.975	*k* = −21→24
12668 measured reflections	*l* = −14→17

### Refinement

**Table:**

Refinement on *F*^2^	Primary atom site location: structure-invariant direct methods
Least-squares matrix: full	Secondary atom site location: difference Fourier map
*R*[*F*^2^ > 2σ(*F*^2^)] = 0.034	Hydrogen site location: inferred from neighbouring sites
*wR*(*F*^2^) = 0.094	H-atom parameters constrained
*S* = 1.06	*w* = 1/[σ^2^(*F*_o_^2^) + (0.0558*P*)^2^ + 0.2342*P*] where *P* = (*F*_o_^2^ + 2*F*_c_^2^)/3
4184 reflections	(Δ/σ)_max_ = 0.003
248 parameters	Δρ_max_ = 0.34 e Å^−^^3^
0 restraints	Δρ_min_ = −0.26 e Å^−^^3^

### Special details

**Table:**

Geometry. All e.s.d.'s (except the e.s.d. in the dihedral angle between two l.s. planes) are estimated using the full covariance matrix. The cell e.s.d.'s are taken into account individually in the estimation of e.s.d.'s in distances, angles and torsion angles; correlations between e.s.d.'s in cell parameters are only used when they are defined by crystal symmetry. An approximate (isotropic) treatment of cell e.s.d.'s is used for estimating e.s.d.'s involving l.s. planes.
Refinement. Refinement of *F*^2^ against ALL reflections. The weighted *R*-factor *wR* and goodness of fit *S* are based on *F*^2^, conventional *R*-factors *R* are based on *F*, with *F* set to zero for negative *F*^2^. The threshold expression of *F*^2^ > σ(*F*^2^) is used only for calculating *R*-factors(gt) *etc*. and is not relevant to the choice of reflections for refinement. *R*-factors based on *F*^2^ are statistically about twice as large as those based on *F*, and *R*- factors based on ALL data will be even larger.

### Fractional atomic coordinates and isotropic or equivalent isotropic displacement parameters (Å^2^)

**Table:**

	*x*	*y*	*z*	*U*_iso_*/*U*_eq_	
S1	0.94253 (4)	1.187575 (15)	0.09160 (2)	0.01899 (10)	
O1	0.66918 (12)	0.79479 (4)	0.06276 (7)	0.02001 (19)	
O2	0.48496 (12)	0.74823 (4)	−0.11902 (7)	0.01879 (19)	
O3	0.40808 (13)	0.83645 (5)	−0.27867 (7)	0.0217 (2)	
N1	0.68396 (15)	1.08768 (5)	−0.14953 (8)	0.0197 (2)	
N2	0.75719 (15)	1.15421 (5)	−0.11050 (8)	0.0215 (2)	
N3	0.80820 (13)	1.07072 (5)	0.01101 (8)	0.0154 (2)	
N4	0.87889 (13)	1.04929 (5)	0.10918 (8)	0.0161 (2)	
C1	0.65260 (15)	0.96291 (6)	−0.08603 (9)	0.0147 (2)	
C2	0.69065 (16)	0.91670 (6)	−0.00221 (9)	0.0157 (2)	
H2	0.7524	0.9338	0.0607	0.019*	
C3	0.63518 (16)	0.84455 (6)	−0.01355 (9)	0.0154 (2)	
C4	0.54233 (16)	0.81924 (6)	−0.10783 (9)	0.0156 (2)	
C5	0.50224 (16)	0.86673 (6)	−0.19093 (9)	0.0161 (2)	
C6	0.55682 (16)	0.93877 (6)	−0.18036 (9)	0.0163 (2)	
H6	0.5298	0.9703	−0.2354	0.020*	
C7	0.74671 (19)	0.82186 (7)	0.16222 (10)	0.0222 (3)	
H7A	0.8675	0.8426	0.1612	0.033*	
H7B	0.7594	0.7830	0.2107	0.033*	
H7C	0.6652	0.8581	0.1814	0.033*	
C8	0.62703 (18)	0.70088 (7)	−0.14319 (11)	0.0233 (3)	
H8A	0.6576	0.7145	−0.2077	0.035*	
H8B	0.5816	0.6521	−0.1470	0.035*	
H8C	0.7369	0.7043	−0.0910	0.035*	
C9	0.36549 (19)	0.88121 (7)	−0.36669 (10)	0.0235 (3)	
H9A	0.2894	0.9210	−0.3527	0.035*	
H9B	0.2987	0.8536	−0.4228	0.035*	
H9C	0.4796	0.8992	−0.3840	0.035*	
C10	0.71354 (16)	1.03844 (6)	−0.07648 (9)	0.0158 (2)	
C11	0.82850 (17)	1.14151 (6)	−0.01460 (10)	0.0175 (2)	
C12	0.95402 (16)	1.10531 (6)	0.15977 (10)	0.0166 (2)	
C13	1.04249 (16)	1.10151 (6)	0.26740 (9)	0.0165 (2)	
C14	1.08199 (17)	1.16412 (7)	0.32607 (10)	0.0205 (3)	
H14	1.0555	1.2091	0.2959	0.025*	
C15	1.15986 (18)	1.15957 (7)	0.42833 (10)	0.0221 (3)	
H15	1.1834	1.2018	0.4663	0.026*	
C16	1.20416 (18)	1.09329 (7)	0.47618 (10)	0.0213 (3)	
C17	1.16536 (18)	1.03064 (7)	0.41664 (10)	0.0221 (3)	
H17	1.1934	0.9857	0.4469	0.026*	
C18	1.08647 (17)	1.03423 (6)	0.31419 (10)	0.0200 (3)	
H18	1.0625	0.9920	0.2762	0.024*	
C19	1.2948 (2)	1.08960 (8)	0.58656 (11)	0.0297 (3)	
H19A	1.4187	1.0700	0.5923	0.044*	
H19B	1.2217	1.0592	0.6226	0.044*	
H19C	1.3021	1.1372	0.6154	0.044*	

### Atomic displacement parameters (Å^2^)

**Table:**

	*U*^11^	*U*^22^	*U*^33^	*U*^12^	*U*^13^	*U*^23^
S1	0.02085 (17)	0.01264 (15)	0.02384 (17)	−0.00229 (10)	0.00504 (12)	−0.00188 (11)
O1	0.0277 (5)	0.0151 (4)	0.0155 (4)	−0.0007 (3)	−0.0008 (4)	0.0016 (3)
O2	0.0206 (4)	0.0136 (4)	0.0223 (5)	−0.0038 (3)	0.0040 (4)	−0.0029 (3)
O3	0.0277 (5)	0.0203 (4)	0.0149 (4)	−0.0055 (4)	−0.0020 (4)	−0.0003 (3)
N1	0.0231 (5)	0.0157 (5)	0.0206 (5)	−0.0005 (4)	0.0047 (4)	0.0002 (4)
N2	0.0271 (6)	0.0150 (5)	0.0233 (6)	−0.0019 (4)	0.0068 (5)	−0.0005 (4)
N3	0.0157 (5)	0.0125 (4)	0.0183 (5)	−0.0004 (4)	0.0042 (4)	0.0001 (4)
N4	0.0158 (5)	0.0161 (5)	0.0164 (5)	0.0004 (4)	0.0030 (4)	−0.0008 (4)
C1	0.0130 (5)	0.0144 (5)	0.0175 (6)	0.0004 (4)	0.0049 (4)	−0.0013 (4)
C2	0.0155 (5)	0.0155 (5)	0.0158 (6)	0.0000 (4)	0.0020 (4)	−0.0020 (4)
C3	0.0154 (5)	0.0148 (5)	0.0160 (6)	0.0016 (4)	0.0030 (4)	0.0009 (4)
C4	0.0150 (5)	0.0137 (5)	0.0186 (6)	−0.0021 (4)	0.0040 (5)	−0.0022 (4)
C5	0.0139 (5)	0.0196 (5)	0.0149 (6)	−0.0012 (4)	0.0028 (4)	−0.0019 (4)
C6	0.0154 (5)	0.0176 (5)	0.0161 (6)	0.0002 (4)	0.0038 (4)	0.0015 (4)
C7	0.0280 (7)	0.0206 (6)	0.0152 (6)	0.0018 (5)	−0.0034 (5)	0.0011 (5)
C8	0.0247 (6)	0.0166 (6)	0.0274 (7)	0.0015 (5)	0.0011 (5)	−0.0029 (5)
C9	0.0249 (6)	0.0302 (7)	0.0146 (6)	−0.0042 (5)	0.0016 (5)	0.0024 (5)
C10	0.0158 (5)	0.0159 (5)	0.0163 (6)	0.0012 (4)	0.0047 (4)	−0.0013 (4)
C11	0.0183 (6)	0.0124 (5)	0.0231 (6)	−0.0007 (4)	0.0069 (5)	0.0001 (4)
C12	0.0151 (5)	0.0138 (5)	0.0223 (6)	−0.0002 (4)	0.0065 (5)	−0.0015 (4)
C13	0.0139 (5)	0.0176 (5)	0.0188 (6)	−0.0012 (4)	0.0052 (5)	−0.0037 (4)
C14	0.0208 (6)	0.0159 (5)	0.0250 (7)	0.0001 (5)	0.0048 (5)	−0.0036 (5)
C15	0.0236 (6)	0.0194 (6)	0.0234 (7)	−0.0032 (5)	0.0047 (5)	−0.0080 (5)
C16	0.0206 (6)	0.0240 (6)	0.0202 (6)	−0.0031 (5)	0.0062 (5)	−0.0042 (5)
C17	0.0266 (6)	0.0177 (6)	0.0227 (7)	−0.0017 (5)	0.0064 (5)	0.0004 (5)
C18	0.0229 (6)	0.0166 (6)	0.0211 (6)	−0.0029 (5)	0.0059 (5)	−0.0038 (4)
C19	0.0365 (8)	0.0299 (7)	0.0217 (7)	−0.0041 (6)	0.0029 (6)	−0.0034 (5)

### Geometric parameters (Å, °)

**Table:**

S1—C11	1.7276 (13)	C7—H7A	0.9600
S1—C12	1.7675 (13)	C7—H7B	0.9600
O1—C3	1.3604 (14)	C7—H7C	0.9600
O1—C7	1.4315 (15)	C8—H8A	0.9600
O2—C4	1.3792 (13)	C8—H8B	0.9600
O2—C8	1.4346 (15)	C8—H8C	0.9600
O3—C5	1.3619 (14)	C9—H9A	0.9600
O3—C9	1.4225 (15)	C9—H9B	0.9600
N1—C10	1.3218 (15)	C9—H9C	0.9600
N1—N2	1.4035 (14)	C12—C13	1.4611 (18)
N2—C11	1.3085 (17)	C13—C14	1.3983 (16)
N3—C11	1.3693 (15)	C13—C18	1.4034 (17)
N3—N4	1.3727 (14)	C14—C15	1.3781 (19)
N3—C10	1.3763 (15)	C14—H14	0.9300
N4—C12	1.3012 (15)	C15—C16	1.3931 (18)
C1—C2	1.3934 (16)	C15—H15	0.9300
C1—C6	1.3935 (17)	C16—C17	1.4044 (17)
C1—C10	1.4655 (16)	C16—C19	1.4981 (19)
C2—C3	1.3959 (16)	C17—C18	1.3812 (18)
C2—H2	0.9300	C17—H17	0.9300
C3—C4	1.3931 (17)	C18—H18	0.9300
C4—C5	1.4017 (16)	C19—H19A	0.9600
C5—C6	1.3915 (16)	C19—H19B	0.9600
C6—H6	0.9300	C19—H19C	0.9600
			
C11—S1—C12	88.01 (6)	O3—C9—H9A	109.5
C3—O1—C7	116.15 (9)	O3—C9—H9B	109.5
C4—O2—C8	113.06 (9)	H9A—C9—H9B	109.5
C5—O3—C9	117.79 (10)	O3—C9—H9C	109.5
C10—N1—N2	109.48 (10)	H9A—C9—H9C	109.5
C11—N2—N1	105.02 (10)	H9B—C9—H9C	109.5
C11—N3—N4	118.38 (10)	N1—C10—N3	108.18 (10)
C11—N3—C10	105.37 (10)	N1—C10—C1	126.06 (11)
N4—N3—C10	136.25 (10)	N3—C10—C1	125.75 (11)
C12—N4—N3	108.12 (10)	N2—C11—N3	111.94 (10)
C2—C1—C6	121.21 (11)	N2—C11—S1	139.02 (9)
C2—C1—C10	120.27 (11)	N3—C11—S1	109.04 (9)
C6—C1—C10	118.52 (11)	N4—C12—C13	122.48 (11)
C1—C2—C3	119.35 (11)	N4—C12—S1	116.44 (10)
C1—C2—H2	120.3	C13—C12—S1	121.08 (9)
C3—C2—H2	120.3	C14—C13—C18	118.77 (11)
O1—C3—C4	115.91 (10)	C14—C13—C12	121.10 (11)
O1—C3—C2	123.93 (11)	C18—C13—C12	120.12 (10)
C4—C3—C2	120.15 (11)	C15—C14—C13	120.43 (12)
O2—C4—C3	120.33 (10)	C15—C14—H14	119.8
O2—C4—C5	119.86 (10)	C13—C14—H14	119.8
C3—C4—C5	119.78 (10)	C14—C15—C16	121.63 (11)
O3—C5—C6	124.79 (11)	C14—C15—H15	119.2
O3—C5—C4	114.71 (10)	C16—C15—H15	119.2
C6—C5—C4	120.50 (11)	C15—C16—C17	117.64 (12)
C5—C6—C1	118.99 (11)	C15—C16—C19	120.81 (11)
C5—C6—H6	120.5	C17—C16—C19	121.52 (12)
C1—C6—H6	120.5	C18—C17—C16	121.46 (12)
O1—C7—H7A	109.5	C18—C17—H17	119.3
O1—C7—H7B	109.5	C16—C17—H17	119.3
H7A—C7—H7B	109.5	C17—C18—C13	120.06 (11)
O1—C7—H7C	109.5	C17—C18—H18	120.0
H7A—C7—H7C	109.5	C13—C18—H18	120.0
H7B—C7—H7C	109.5	C16—C19—H19A	109.5
O2—C8—H8A	109.5	C16—C19—H19B	109.5
O2—C8—H8B	109.5	H19A—C19—H19B	109.5
H8A—C8—H8B	109.5	C16—C19—H19C	109.5
O2—C8—H8C	109.5	H19A—C19—H19C	109.5
H8A—C8—H8C	109.5	H19B—C19—H19C	109.5
H8B—C8—H8C	109.5		
			
C10—N1—N2—C11	0.10 (13)	C2—C1—C10—N1	−179.02 (11)
C11—N3—N4—C12	0.36 (14)	C6—C1—C10—N1	1.10 (18)
C10—N3—N4—C12	−179.17 (12)	C2—C1—C10—N3	−0.50 (18)
C6—C1—C2—C3	1.35 (17)	C6—C1—C10—N3	179.61 (10)
C10—C1—C2—C3	−178.53 (10)	N1—N2—C11—N3	0.69 (13)
C7—O1—C3—C4	−173.70 (10)	N1—N2—C11—S1	−179.31 (11)
C7—O1—C3—C2	7.59 (16)	N4—N3—C11—N2	179.15 (9)
C1—C2—C3—O1	178.51 (10)	C10—N3—C11—N2	−1.19 (13)
C1—C2—C3—C4	−0.15 (17)	N4—N3—C11—S1	−0.85 (13)
C8—O2—C4—C3	−87.14 (13)	C10—N3—C11—S1	178.81 (7)
C8—O2—C4—C5	94.73 (13)	C12—S1—C11—N2	−179.22 (15)
O1—C3—C4—O2	2.14 (16)	C12—S1—C11—N3	0.79 (9)
C2—C3—C4—O2	−179.09 (10)	N3—N4—C12—C13	179.87 (10)
O1—C3—C4—C5	−179.73 (10)	N3—N4—C12—S1	0.32 (12)
C2—C3—C4—C5	−0.96 (17)	C11—S1—C12—N4	−0.68 (10)
C9—O3—C5—C6	1.35 (17)	C11—S1—C12—C13	179.76 (10)
C9—O3—C5—C4	−178.93 (10)	N4—C12—C13—C14	−164.70 (11)
O2—C4—C5—O3	−0.68 (16)	S1—C12—C13—C14	14.83 (16)
C3—C4—C5—O3	−178.82 (10)	N4—C12—C13—C18	14.20 (17)
O2—C4—C5—C6	179.05 (10)	S1—C12—C13—C18	−166.27 (9)
C3—C4—C5—C6	0.91 (17)	C18—C13—C14—C15	−1.05 (18)
O3—C5—C6—C1	179.96 (11)	C12—C13—C14—C15	177.87 (11)
C4—C5—C6—C1	0.26 (17)	C13—C14—C15—C16	0.95 (19)
C2—C1—C6—C5	−1.40 (17)	C14—C15—C16—C17	−0.48 (19)
C10—C1—C6—C5	178.49 (10)	C14—C15—C16—C19	177.87 (12)
N2—N1—C10—N3	−0.83 (13)	C15—C16—C17—C18	0.14 (19)
N2—N1—C10—C1	177.90 (11)	C19—C16—C17—C18	−178.20 (12)
C11—N3—C10—N1	1.20 (13)	C16—C17—C18—C13	−0.27 (19)
N4—N3—C10—N1	−179.23 (11)	C14—C13—C18—C17	0.71 (17)
C11—N3—C10—C1	−177.54 (11)	C12—C13—C18—C17	−178.21 (11)
N4—N3—C10—C1	2.0 (2)		

### Hydrogen-bond geometry (Å, °)

**Table:**

*D*—H···*A*	*D*—H	H···*A*	*D*···*A*	*D*—H···*A*
C2—H2···N4	0.93	2.37	3.059 (2)	131
C6—H6···N1	0.93	2.61	2.913 (2)	100
C14—H14···S1	0.93	2.72	3.131 (2)	108
C7—H7B···O3^i^	0.96	2.45	3.202 (2)	135

Symmetry codes: (i) *x*+1/2, −*y*+3/2, *z*+1/2.
